# Metabolic Biomarkers in B-Cell Lymphomas for Early Diagnosis and Prediction, as Well as Their Influence on Prognosis and Treatment

**DOI:** 10.3390/diagnostics12020394

**Published:** 2022-02-03

**Authors:** Abdullah Alfaifi, Salem Bahashwan, Mohammed Alsaadi, Hafiz Malhan, Aqeel Aqeel, Waiel Al-Kahiry, Hussein Almehdar, Ishtiaq Qadri

**Affiliations:** 1 Department of Biological Science, Faculty of Science, King AbdulAziz University, Jeddah 21589, Saudi Arabia; aalfaifi1@moh.gov.sa (A.A.); maalsaadi1@kau.edu.sa (M.A.); hmehdar@kau.edu.sa (H.A.); 2 Fayfa General Hospital, Ministry of Health, Jazan 83581, Saudi Arabia; 3 Hematology Research Unit, King Fahad Medical Research Center, King AbdulAziz University, Jeddah 21589, Saudi Arabia; smbahashwan1@kau.edu.sa; 4 Department of Hematology, Faculty of Medicine, King AbdulAziz University, Jeddah 21589, Saudi Arabia; 5 King AbdulAziz University Hospital, King AbdulAziz University, Jeddah 21589, Saudi Arabia; 6 Prince Mohammed Bin Nasser Hospital, Ministry of Health, Jazan 82943, Saudi Arabia; dr-hafiz@hotmail.com (H.M.); amaqeel@moh.gov.sa (A.A.); walkahiry@moh.gov.sa (W.A.-K.)

**Keywords:** B-cell lymphoma, metabolism, prognosis, biomarkers, therapeutic targets

## Abstract

B-cell lymphomas exhibit a vast variety of clinical and histological characteristics that might complicate the diagnosis. Timely diagnosis is crucial, as treatments for aggressive subtypes are considered successful and frequently curative, whereas indolent B-cell lymphomas are incurable and often need several therapies. The purpose of this review is to explore the current advancements achieved in B-cell lymphomas metabolism and how these indicators help to early detect metabolic changes in B-cell lymphomas and the use of predictive biological markers in refractory or relapsed disease. Since the year 1920, the Warburg effect has been known as an integral part of metabolic reprogramming. Compared to normal cells, cancerous cells require more glucose. These cancer cells undergo aerobic glycolysis instead of oxidative phosphorylation to metabolize glucose and form lactate as an end product. With the help of these metabolic alterations, a novel biomass is generated by the formation of various precursors. An aggressive metabolic phenotype is an aerobic glycolysis that has the advantage of producing high-rate ATP and preparing the biomass for the amino acid, as well as fatty acid, synthesis needed for a rapid proliferation of cells, while aerobic glycolysis is commonly thought to be the dominant metabolism in cancer cells. Later on, many metabolic biomarkers, such as increased levels of lactate dehydrogenase (LDH), plasma lactate, and deficiency of thiamine in B-cell lymphoma patients, were discovered. Various kinds of molecules can be used as biomarkers, such as genes, proteins, or hormones, because they all refer to body health. Here, we focus only on significant metabolic biomarkers in B-cell lymphomas. In conclusion, many metabolic biomarkers have been shown to have clinical validity, but many others have not been subjected to extensive testing to demonstrate their clinical usefulness in B-cell lymphoma. Furthermore, they play an essential role in the discovery of new therapeutic targets.

## 1. Introduction

Lymphomas are solid immune system tumors. Non-Hodgkin’s lymphoma makes up 90% of lymphomas, while the other 10% is marked by Hodgkin’s lymphoma [1]. NHL or non-Hodgkin’s lymphomas are a diverse category of cancers, with B-lymphocytes accounting for 85–90% of cases and T-lymphocytes or NK-lymphocytes accounting for the remaining 10% [1,2,3]. Based on the immunophenotypical, morphological, clinical, etiological, and genetic evidence, the World Health Organization (WHO) in 2016 classified the lymphoid neoplasm and differentiated about eighty types of non-Hodgkin’s lymphomas. A violent form of B-cell lymphoma is diffuse large B-cell lymphoma, henceforth referred to as DLBCL, which is considered as the most common form of all B-cell lymphomas, while follicular lymphoma (FL) is considered as the most common mild form of B-cell lymphomas, which account for 35% and 20%, respectively [4]. These different subtypes of B-cell lymphomas usually arise in the spleen, lymph nodes, or bone marrow but can also develop in almost any extra-lymphatic organ or tissue [1,5].

The proliferation of human cells is not independent; indeed, the entry of normal cells into the cell cycle is triggered only when well-controlled instructions are given by either the cell itself or the chemical signaling, neighboring cells, and the microenvironment. The noncancerous, or the normal, cells interact with the respective receptor by a stimulus that triggers extracellularly (e.g., growth factor) to enhance the signaling sequence within the cell (intracellular), which, in turn, results in the duplication of cells. In contrast, in tumor cells, the number of growth factor receptors increases with genetic mutations. The proliferation of the cell is encouraged by signaling pathways occurring within the cell (intracellular), which can continuously be amplified or enhanced—hence, enabling the development and proliferation of malignant cells by themselves [6]. Contrary to normal cells, for meeting the high demand of generating power and building blocks, the rearrangement of cellular metabolic pathways is done by cancerous cells to enhance cell growth [7,8,9].

**Figure 1:**
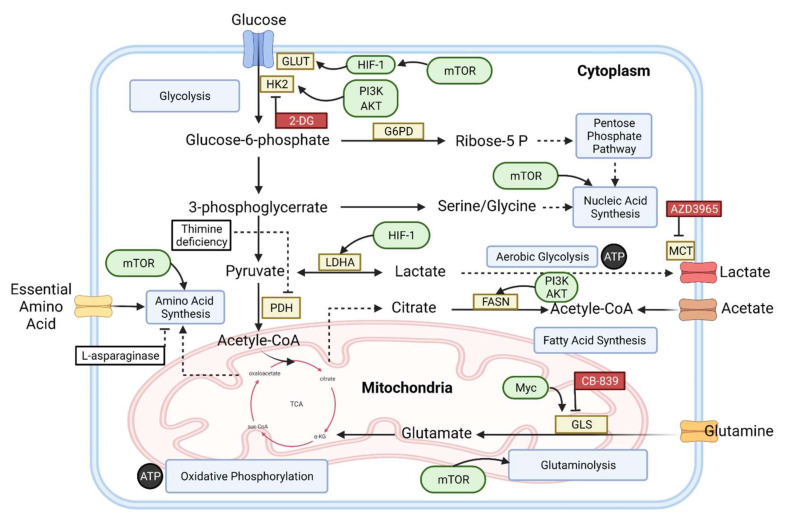
Metabolic pathways associated with B-cell lymphoma [12,13,14,15,16,17].

The living cells undergo a series of complicated biochemical procedures called metabolism for producing energy and sustaining their survival. The formation and breaking down of glucose, amino acids, and fatty acids, along with oxidative phosphorylation and ATP production (energy), are all a part of metabolism (Figure 1) [10]. In different types of cancers, metabolomic approaches have been used to classify potential markers and primary metabolic pathways. When compared to normal differentiated cells, proliferating cancer cells have a distinct metabolic profile [11].

Biomarkers are useful in confirming malignant or benign diseases with asymptomatic subjects in the early stages of cancer and in patients examined for neoplastic diseases. Biomarkers can be predictive and helpfully selected for the treatment program when diagnosed with malignant diseases [18].

In diagnosing and managing cancer patients, biomarkers play a key role and are important in meeting the oncological precision promise. Despite the fact that many biomarkers have been shown to have clinical validity, many others have not undergone thorough testing to demonstrate their clinical usefulness, preventing them from being properly integrated into clinical treatment [19]. This review aims to discuss the latest progress made in lymphoma metabolism, focusing, in particular, on biomarkers of metabolites and how these markers contribute to early B-cell lymphomas diagnosis.

## 2. Basic Metabolism in Normal Cells

Metabolic pathways that generate energy in biological systems are necessary for cells to proliferate and survive. This energy is acquired by the oxidation of macromolecules such as carbohydrates, proteins, and lipids that make up the living body’s chemical composition. Glycolysis is a metabolic pathway that initially appears in carbohydrate metabolism. It occurs in all cells. Oxidative phosphorylation (OXPHOS), which occurs in the presence of oxygen in cells containing mitochondria and is capable of producing far more energy than glycolysis, acts as an active electrical factory inside our cells [20]. Glycolysis turns one glucose molecule into two pyruvate molecules. Glycolysis is a ten-step reaction that earns eight ATPs during aerobic glycolysis. The conversion of pyruvate into lactate takes place in conditions that are anaerobic in nature (e.g., muscle cells). This transition is catalyzed by the lactate dehydrogenase-A (LDH-A) enzyme. This enzyme belongs to the oxidoreductases group. LDH-A is a key metabolic enzyme that guarantees that the oxidized form of nicotinamide adenine dinucleotide (NAD+) is continuously delivered to cells, and lactate is subsequently expelled by monocarboxylate transporters into extracellular space (MCTs) [6,21]. Thus, each glucose molecule generates two ATPs during anaerobic glycolysis.

In comparison to glycolysis, oxidative phosphorylation produces a significant amount of energy in the presence of oxygen in cells containing mitochondria. Proteins, which are large complex macromolecules, are hydrolyzed to form amino acid structures, carbohydrates to form monosaccharide structures, and fats to form glycerol and fatty acid structures. These building components are introduced into the tricarboxylic acid cycle (TCA cycle, also called the Krebs’ Cycle) through the two-carbon molecule acetyl CoA. The oxidation of acetyl CoA to CO_2_ is also a part of this cycle. The last stage of the TCA cycle is the transfer of electrons from FADH_2_ and NADH to the electron transfer chain. At the end of the cycle, the OXPHOS pathway produces 36 ATPs. Glycolysis and OXPHOS, therefore, work together to maintain the energy balance of cells [20,22].

## 3. Metabolic Alteration in B-Cell Lymphoma

Cancer metabolic alterations include cellular changes related to carcinogenesis, which is the cancer formation process. While oncogenic signaling pathways are active, the circumstances for cancer cell survival are also provided. The alterations that occur enable the tumor cells to grow and proliferate, and this modification promotes the tumor cells in maintaining the environment required for survival. The usage of glucose and lactate generation greatly increased in cancer cells and other proliferating cells, including B-cell lymphoma. During this process, NAD+ are rapidly added to the pool. This alteration, called the “Warburg Effect”, has been programmed to enhance the uptake of glucose, as well as glycolysis, in cancerous cells, despite the presence of oxygen in the environment [23,24]. The presence of oxygen inhibits glycolysis in mammalian cells. Louis Pasteur coined the term “Pasteur effect” to describe how the glucose flow decreases in aerobic conditions. The Warburg effect, also called aerobic glycolysis, is the process by which glucose forms lactic acid in aerobic conditions in cancer cells. Cancer cells with the Warburg phenotype shift to aerobic glycolysis from oxidative phosphorylation for energy consumption [25]. By contrast, cancer cells continue to employ glycolysis in aerobic conditions. Tumor glycolysis is referred to as the “Warburg effect” or the “aerobic glycolysis” to differentiate it from normal anaerobic glycolysis. Due to the influence of the Warburg effect, cancerous cells are programmed to enhance the uptake of glucose, as well as glycolysis. As a consequence, the expression of GLUT receptors, which are glucose transporters, increases, allowing more glucose into the cell to perform energy regulation [13,26]. Aerobic glycolysis is commonly thought to be the dominant metabolism in cancer cells, and new research indicates that cancer cells also use mitochondrial OXPHOS [27].

The constant increase in glycolysis results in acute and chronic acidity of the tumor microenvironment. Tumor cell groups survive living in this acidic environment and grow. The glycolytic phenotype of cancer cells is required for invasive tumor development, and this glycolytic phenotype promotes malignant development by enhancing the cancer cell potential. Cancer cells require a lot of glucose and glutamine. Cancer cells need modulators such as glucose and glutamine. Due to these modulators, it can produce the proteins and lipids it requires. They utilize the fundamental cell processes such as the tricarboxylic acid cycle, OXPHOS, and the pentose phosphate pathway to carry out these synthesis reactions. These mechanisms provide enough metabolites to stimulate cell growth. The pentose phosphate pathway (PPP) is important, because it utilizes the intermediate pathway of glycolysis. In this pathway, glucose molecules are acquired for nucleic acids. Increased biosynthetic activity in the cell results in an increase in NADPH production, which helps maintain the cell’s redox balance [28]. Even though the glycolysis occurring in the presence of oxygen is not very efficient, it produces more ATP and forms important polymers like amino acids, carbons, and nucleotides for the synthesis of lipids or fatty acid (Figure 1), all of which are required for cell division [29,30]. Typically, tumor cells have significantly increased intakes of glucose and glycolytic rates. More intermediate glycolytic metabolites are produced, resulting in more ATP being generated. Furthermore, a large fraction of glucose is shunted into glycolytic pathways instead of being utilized for the production of pyruvate. Finally, with the help of the enzyme LDH present in the cytoplasm, almost all the pyruvate gets converted into lactate and secreted after glycolysis instead of getting into the process of oxidation by mitochondrial metabolism. This process happens even when the oxygen is present in a surplus amount for supporting respiration occurring in mitochondria (Figure 1). Recent studies have correlated the increased level of LDH and plasma lactate concentration to the increased mortality rate of B-cell lymphoma patients [31]. In a MYC-transformed Burkitt lymphoma (BL) model, inhibiting LDH led to tumor reduction in vivo [32]. Lactic acidosis is described by the Warburg effect as a process that occurs as a result of the disruption of lactate homeostasis because of both underutilization and overproduction. There are two types of lactic acidosis: type A and type B. The first one, i.e., Type A, is caused by the lack of oxygen in the cells. For type B lactic acidosis occurring in normoxic cells, other factors such as toxins or drugs are responsible instead of oxygenation [33]. The second type of lactic acidosis, i.e., Type B, is caused by changes in the glycolytic processes and their redox effects. Many human cancers, especially lymphomas and leukemias, have Type B lactic acidosis [34,35]. Lactic acidosis causes thiamine deficiency, which is a distinguishable feature associated with it. Thiamine acts as a cofactor that helps pyruvate dehydrogenase convert pyruvate to acetyl-CoA. When thiamine is absent in malignant cells, the rapid conversion of pyruvate into lactate takes place. Lactic acidosis has an insignificant prognosis if the treatment is delayed [36,37].

## 4. An Overview of Metabolic Biomarkers in B-Cell Lymphomas

According to the National Institute of Health (NIH) Biomarkers Definitions Working Group, a biomarker is defined as a characteristic that is evaluated and measured as a sign for normal pathogenic, biological, or pharmacological processes to remedial interventions. In order to examine normal biological processes, biomarkers are often measured and assessed; distinct biological indicators of a process or status can be reliably measured in body tissue, cells, or fluids and can be used in the early detection of health changes in a patient. The biomarkers can be employed to examine how well the body responds against cancer or any other disease [19]. Biomarkers are commonly used for medical purposes to provide prognostic or predictive information [38]. Biomarkers are driven from the measurements of metabolites, proteins, or DNA frequently in blood samples in clinical medicine, because blood analysis is low-cost and easy to extract from patients [39]. Any sort of intracellular signaling pathways that use cell energetic needs and support cell division or cell viability or both is termed as metabolism. To perform better and more effectively, the T- and B-lymphocytes and all the mammalian cells alter their metabolic programs, along with phenotypic changes. Exacerbated metabolism and metabolic flexibility are biomarkers of tumorigenesis and tumor cell progression in a complex tumor microenvironment [20]. To help understand the metabolic processes in cells, organs, and organisms, a new technique has been evolved, which is termed metabolomics. For all quantitative analyses, metabolomics focuses on numerous metabolites that represent the protein functions and, also, the end products of genes and transcripts. Metabolomics emphasizes greatly the fact that gene abundance and transcriptional and protein dynamics can have a substantial impact on metabolite quantity and dynamics [40]. The differences in the lymphoma patients’ metabolic fingerprints are the keys to finding and developing specific biomarkers [41].

### 4.1. Glucose Uptake

Increased lipid metabolism and high glucose uptake are shown by malignancies originated by B cells [42]. Lymphoma patients are diagnosed by the imaging of metabolism tumor cells by using 18 Fluro deoxyglucose positron emission tomography (18FDG-PET). FDG-PET is critical for determining the initial extent of the disease, the therapeutic response, the prognosis, and the treatment decisions in some subtypes [43,44]. Many researches show that aggressive or, commonly known as, high-grade B-cell lymphomas are more addicted to FDH than low-grade or indolent B-cell lymphomas [45]. Grades 2 and 3 follicular lymphoma and diffuse large B cells exhibit the highest FDH metabolisms. In DLBCL, a high glucose uptake is thought to be a potential biomarker of an aggressive tumor and is correlated with a bad prognosis [46]. In DLBCL patients, early FDG-PET cannot predict the prognosis, as found by many previous studies. However, the initial response of metabolism-targeted medication can be monitored by FDG-PET as a pharmacodynamic marker. A recent clinical study of B-cell lymphomas stated that early FDG-PET has great significance in catching any metabolic changes in the glucose level associated with mTOR inhibitor treatment. Since mTOR inhibition reduces cell lactatogenicity, some surrogate pharmacodynamic markers like magnetic resonance spectroscopy are evolved to monitor the intratumoral lactate concentrations [47].

### 4.2. CHK α

Phosphocholine (P-Cho), total choline (tCho), and phosphatidylcholine (PC) are markers of carcinogenesis metabolism and/or tumor development. With over 50% of all phospholipids in mammalian cells and organelles, phosphatidylcholine is the most prevalent phospholipid. Modifications in enzymes controlling the catabolic and anabolic processes of phosphatidylcholine, for example, PC-specific phospholipase-C (PC-PLC), choline kinase-α (CHK α), glycerophosphocholine phosphodiesterases (GPCPDs), and PC-PLD1, are sometimes linked with the regulatory activity and actions of various choline transporters. The analyses conducted on the metabolomic studies of plasma or serum obtained from animal models and patients suffering from lymphoid malignancies also indicate changes in the metabolism of choline that also include decreased levels of plasma and serum choline, LPC, GPC, and PC [48].

On the other hand, the initial images obtained from MRS investigations pointed out that the aberrant metabolism of choline may be detected in non-Hodgkin’s lymphoma (NHL) and central nervous system lymphoma (CNSL) human and animal models. The images obtained from positron emission tomography (PET-CT) using choline biomarkers has recently emerged as a viable method for presenting and monitoring the response occurring after therapy in such patients who are suffering from lymphoid malignant tumors, with greater specificity and sensitivity for CNSL and multiple myeloma (MM) than the commonly employed sugar (glucose) uptake [49].

Therefore, the metabolomic data, as well as imaging, have demonstrated that lymphoid malignancies have enhanced the uptake of choline and a higher activity of the choline kinase enzyme. It is necessary to conduct additional research on the key enzymes involved in metabolism that regulate both the catabolic and anabolic pathways of PC in lymphoid malignancies in order to gain better knowledge about lymphomagenesis and, also, the development of better therapeutic and diagnostic interventions for lymphoid malignancies [50].

### 4.3. LDH and β2-MG

LDH has been known to have an important role in the metabolism of tumors. Another metabolic process, in which cancerous cells depend on anaerobic (without oxygen) respiration as their primary energy source, is a characteristic of most cancer cells. Even when oxygen is present in sufficient amounts, lactate is formed from glucose (Warburg effect). As a result, tumor cells consume excess glucose and use it to speed up their development and proliferation [51]. Many cancer patients have elevated serum LDH activity, which can be used as a prognostic indicator [31,52]. The LDH levels are significant in patients with aggressive B-cell lymphoma, which can be employed as a screening tool for knowing the response of a particular treatment or its recurrence. Emphasis should also be given to some additional parameters, such as the levels of LDH for evaluating the prognosis of patients with B-cell lymphoma. Unfortunately, the serum levels of LDH are only important for prognosis and are not particular for diagnosing cancer [31]. In a study on patients with B-cell lymphoma, researchers looked at the importance of beta-2 microglobulin (β 2-MG) and LDH. They proved that elevated serum levels of both β 2-MG and LDH have a clinical importance in patients with a B-cell lymphoma condition, its prognosis, and short-term therapeutic response [53]. We conclude that, by combining several biomarkers, we may accurately predict the response to therapy in B-cell lymphoma patients.

### 4.4. NEK2

Serine/threonine kinase NIMA-related kinase 2 (NEK2) belongs to the family of NIMA-related kinase (NEK) that is involved in cell cycle regulation and is also overexpressed in a number of tumors, such as DLBCL. Previously published information indicates that the overexpression of NEK2 in a variety of human tumors is strongly associated with tumor progression processes such as chromosomal instability, excessive proliferation, treatment tolerance, and metabolism. Yet, NEK2’s significance and underlying processes in B-cell lymphoma have rarely been studied [54]. Gu Z et al. observed that NEK2 promoted the unprompted development of the germinal center and boosted T-cell-dependent immunological responses [55]. In the process of glycolysis, a rate-limiting enzyme called pyruvate kinase (PK) helps in regulating the last stage by initiating an irreversible conversion of PEP into pyruvate and producing ATP. The expression of mammalian pyruvate kinase isomer 2 (PKM2) is extensive in quickly dividing cells, including tumor cells and embryos. PKM2 is a kinase involved in metabolic reprogramming and a transcriptional coactivator engaged in the proliferation of tumor cells and metastasis. Posttranslational changes, particularly phosphorylation, influence PKM2 expression and function. Zhou et al. revealed that the expression of NEK2 was substantial in DLBCL and was overexpressed with a poor prognosis in patients with DLBCL. The functional cell growth of DLBCL was enhanced by NEK2, as it promotes aerobic glycolysis through PKM2. This marker offers a new concept for treating DLBCL patients with targeted treatment [55].

### 4.5. Glutamine

MYC regulates glycolysis and mitochondrial respiration [56]. When MYC is high, genetic alterations enhance the glucose intake and lactate generation. Additionally, the hypoxic- and glucose-deficient areas seen in solid tumors need the use of other survival and/or growth strategies. The glucose-independent pathway allows cancer cells to be alive and multiply in the condition of hypoxia and glucose deprivation present in the tumor microenvironment. For cell survival and proliferation, the metabolism of glutamine may maintain the TCA cycle when glucose is absent [14]. Glutamine offers energy for cancer cells to build macromolecules. Even when oxygen is present, cancer cells acquire energy (ATP) through phosphorylation and glycolysis. Glucose and glutamine are essential substrates for metabolism and propagation, as well as tumor growth. Thus, for the generation of ATP through glycolysis, glucose is used, whereas, for the synthesis of TCA in mitochondria, NADPH, and fatty acid, glutamine is used [57]. On the other hand, metabolic inhibitors have varied effects on B-cell lymphomas with various features. The inhibitor of the enzyme glutaminase, i.e., BPTES (bis-2-(5-phenylacetimido- 1,2,4, thiadiazol-2-yl) ethyl sulfide) can target cancer cells because of the critical role the glutamine metabolic pathway plays in cell proliferation and survival under hypoxic conditions, as well as the deprivation of glucose. Previous studies looked at whether hypoxia-induced oxidative stress might be induced by inhibiting the metabolism of glutamine. When glutaminase (GLS) gets inhibited by the selective inhibitor of glutaminase (BPTES), the oxygen-deprived cells show elevated ROS and decreased levels of ATP. In fact, they discovered that the in vivo inhibition of glutaminase efficiently suppresses the growth of hypoxic cancerous cells and slows down the in vivo tumor xenograft development. This novel remedial approach to target tumor metabolism on the basis of metabolic characteristics may be fruitful for B-cell lymphoma [14,58].

### 4.6. HK2

Lymphoid organs are hypoxic and have a lesser concentration of oxygen as compared to blood. B cells are found in the bone marrow. The bone marrow has a lower concentration of oxygen (pO_2_ 1.3%) and an oxygen concentration of 0.6–2.8% in extravascular tissues. Some secondary lymphoid tissues, like the spleen, also have B cells, where they also get exposed to low oxygen concentrations at the levels of 0.5–4.5% pO_2_. As a result, B cells present in the entire body experience different oxygen levels. The core part of the tumor microenvironment has a low oxygen concentration (hypoxic), exerting a significant influence on a variety of metabolic pathways. Under hypoxia, two kinases, PERK and mTORC, are required for the repression of protein synthesis. During hypoxia, mTORC-dependent eIF4E sequestration inhibits cap-mediated translations [59]. Tumor cell metabolism is reprogrammed to maximize the glucose consumption for providing a source to synthesize the amino acids, lipids, and nucleotides essential for tumor development. The first step in glucose metabolism is done by the hexokinase II (HK2) enzyme. In aggressive tumors, this enzyme is overexpressed. Glucose gets phosphorylated to glucose-6-phosphate (G6P) by HK2. The cells subsequently use it through the chief glucose metabolism pathways, including glycolysis, PPP metabolism, and glycogenesis, to fulfill the metabolic demands of a developing tumor. Numerous B-cell malignancies have enhanced glucose absorption and lipid metabolism. The cases of B-cell aggressive lymphoma revealed the gene expression profiles of lipogenic pathways. They pointed out the substantially elevated levels of adipophilin in individuals with Burkitt lymphoma (BL), indicating the use of adipophilin as a metabolic target for diagnosing BL [60].

In 2018, Bhalla et al. used molecular profiling to study the targets of inducible factor-1 alpha (HIF1) in the prognosis of patients with DLBCL. They demonstrated that activating HIF1 causes protein translation. The increased expression of hypoxia-related targets like HK2, CYT-C, and GLUT1 has been located. The suppression of translation resulted in a reduction in mitochondrial function. Additionally, genetic knockout experiments have shown that HK2, the rate-limiting enzyme in glycolysis, is essential to accelerate DLBCL development in the presence of hypoxic stress. The result of Bhalla et al. implies that HK2 has a direct role in the development of B-cell lymphoma and is a significant metabolic handler of the DLBCL phenotype [59].

### 4.7. Notch2

The NOTCH pathway regulates key cell fate choices throughout embryonic development. Physiologically, NOTCH family signaling promotes proliferation of the cells, necrosis (death of the cell), and the process of cell differentiation. The signals mediated by NOTCH are the key controllers of self-renewal in adult tissues, including myogenesis, neurogenesis, and lymphocyte development. The functions and components of NOTCH signaling have been related to a number of malignancies, such as hematological malignancies and solid tumors, where NOTCH may serve as a tumor suppressor or an oncogene. The gain-of-function mutations of NOTCH1 and NOTCH2 have been testified in B-cell lymphomas. NOTCH activation by non-mutational mechanisms has been observed in MM [61]. NOTCH2 mutations in DLBCL were one of the first NOTCH pathway gene alterations found in mature B-cell neoplasms [62]. Following this, NOTCH2 mutations were identified in 6–8% of DLBCL. NOTCH1 mutations were identified in 7 to 8% of DLBCL patients. That study found more common (12%) mutations in DTX1, another NOTCH signaling protein. DTX1 N-terminal domains directly engage with the NICD and suppress NOTCH signaling by inhibiting transcriptional coactivator recruitment. Due to this, certain DTX1 mutants have increased NOTCH signaling [63]. Mutations in NOTCH1 and NOTCH2 are linked with a poor prognosis in the majority of mature B-cell malignancies in both prospective and retrospective clinical studies [61]. However, more research is needed to determine whether or not NOTCH2 has any clinical significance in the prediction, diagnosis, or prognosis of B-cell lymphomas.

### 4.8. Metabolic Profile

Metabolomics research reveals intriguing variations among lymphoma patients, enabling pathological variations from healthy individuals to be distinguished. The observed metabolic trends may serve as early biomarkers of lymphoma, although further research is required to confirm this [40]. A metabolomics method has recently been suggested to discover biomarkers with the potential for characterizing and diagnosing various subtypes of lymphomas at the earliest time [64].

#### 4.8.1. Hypoxanthine and Elaidic Acid

Barberini et al. investigated the use of the GC-MS method to evaluate plasma samples collected from patients with various lymphoma subtypes. The patients were from frequency-matched age (10-year groups) and gender populations in the research. The study’s goal was to find potential metabolic biomarkers for early detection and differential diagnosis between the various subtypes of lymphomas [40]. Hypoxanthine and elaidic acid were shown to be more prevalent in the patients with DLBCL, CLL, MM, and HL (Hodgkin’s lymphoma) than in the healthy controls in all four models. Hypoxanthine is a purine essential in the metabolism of adenine and guanine, as well as in the production of their nucleosides. Elaidic acid is a trans isomer of monounsaturated C18 oleic acid found naturally in ruminant fat, meat, margarine, and baked goods [65]; its plasma level has been linked to an increase in the total mortality and cardiovascular mortality, and a diet high in trans fatty acids has been linked to an increased risk of B-cell lymphomas. For the first time, they show that lymphoma patients have higher levels of elaidic acid plasma [40]. There are many limitations to this research, including the small sample size and focus on children as the only age group included. However, since abnormal metabolic pathways are found as early biomarkers of lymphoma, further research is needed.

#### 4.8.2. B Vitamins

The nutrition status was identified as a key predictive prognostic predictor in the international prognostic index (IPI). A mechanism that is required for humans to take nutrients is one-carbon (OCM) metabolism. Both B vitamins serve directly as the folate supply to the single-carbon unit or indirectly as key OCM path coenzymes, including B2 vitamin (riboflavin), B6 vitamin (pyridoxal 50-phosphate), and B12 vitamin (cobalamin) [66]. B6 and B12 are important sources of coenzymes actively involved in the OCM process. Folate enters the cell membranes by means of the folate receptor and is reduced to dihydrofolate and, then, to THF in order to synthesize OCM coenzymes. Additionally, vitamin B2 is critical to nutrition metabolism and is actively involved in anti-inflammatory response control [67]. Cao et al. studied the clinical significance of B vitamins and single-nucleotide polymorphisms (SNPs) to DLBCL genes in a cohort study, and it was discovered that changes in B vitamin metabolism influenced disease development and had a prognostic impact on DLBCL.

### 4.9. Genetic Alterations

An alteration in metabolism has long been recognized as a key mechanism in tumorigenesis, making it a biomarker of cancer [68]. B-cell lymphoma frequently has abnormal mTORC1 activation, which reprograms multiple metabolic pathways such as nucleotide synthesis, amino acid synthesis, fatty acid synthesis, and glutaminolysis. Furthermore, MYC is a key inducer of many genes involved in anabolic growth, such as transporters and enzymes involved in glycolysis, mitochondrial biogenesis, fatty acid synthesis, and glutaminolysis [12,13,69]. MYC is a gene involved in cellular proliferation that has been found to be dysregulated in B-cell lymphomas [70]. MYC and hypoxia-inducible factor 1 (HIF-1) reprogramming in malignant tissues allows them to better survive tumor microenvironmental alterations. These genes can influence one another; for example, mTOR can activate HIF-1 expression even in normoxic conditions (Table 1) [71].

#### 4.9.1. PI3K/AKT/mTOR

Recent research has emphasized that the aberrant expression of the phosphatidylinositol 3-kinase (PI3K)/AKT/mammalian target of the rapamycin (mTOR) pathway plays a crucial role in tumor cell proliferation and survival, including B-cell lymphomas [84,85,86]. The PI3K/AKT pathway interacts with the intricate molecular mechanism that regulates cellular energy and glucose metabolism [87]. Somatic alterations in specific nodes of the pathway and activation by receptor tyrosine kinases (RTKs) are the two main mechanisms of PI3K/AKT activation in solid tumors [87]. Protein kinase AKT played an important role in regulating the growth of a tumor, metabolic responses, cell proliferation, migration, and apoptosis. In many human cancers, a constituent activation of these protein kinases was mainly involved through phosphorylation [88,89,90]. phospho-AKT (p-AKT) could be a biomarker predictive of clinical outcomes and the response to inhibitors of the mTOR pathway [91]. AKT-mediated phosphorylation may affect the activity of proteins such as caspase-9, some Bcl-2 family members, NF-κB, and other transcription factors that either initiate or inhibit apoptosis. The deregulation of PI3K/Akt can also contribute to tumorigenesis, metastasis, and chemotherapy resistance [92,93]. A study was conducted on 100 patients with DLBCL in King Faisal Specialist Hospital and Research Centre in KSA. The study’s findings established that, in human DLBCL cell lines, the PI3K/AKT pathway is constitutively activated. The inhibition of PI3K induces apoptosis in the majority of DLBCL cells via mitochondrial cytochrome c release and the activation of downstream caspases. Additionally, patients with high p-AKT expression had a poor prognosis. Therefore, not only p-AKT expression can be used for prognosis, but also, these findings may pave the way for further research into the efficacy of a new approach for treating DLBCL using inhibitors of the PI3K/AKT pathway [84]. Metabolic reprogramming is primarily regulated in cancer cells by the serine/threonine kinase mTOR. The mTOR complex 1 detects environmental changes and arranges cellular responses to maintain and function cells [94]. When cells face environmental changes (variability in growth factors, nutrients, oxygen, and immune signals), the mTOR activity is reduced to conserve ATP for anabolic processes such as nucleotide, lipid, and protein synthesis. Growth factor receptors send growth factor signals to the PI3K/Akt pathway. Activated Akt phosphorylates the tuberous sclerosis complex (TSC) protein TSC2 at multiple sites, inactivating the heterotrimeric TSC (which includes TSC1, TSC2, and TBC1D7), a critical regulator of the small G protein Ras homology enriched in brain (Rheb) GTPase. Growth factors and amino acids are required for the activation of mTORC1 at the lysosome. Inactivation of the TSC complex by Akt leads to the release of TSC from Rheb to become Rheb-GTP and activates mTORC1. Both TSC and Rheb are at the surface of the lysosomes. Via the phosphorylation of TSC1 or TSC2, the inflammation-activated kinase, the NF-κB regulator IκBα kinase (IKKβ), or other growth factor-activated kinases (Ras/Raf/MEK/ERK) converge on TSC complex inhibition and mTORC1 activation, respectively. Energy stress or hypoxia, on the other hand, activates TSC by inducing the metabolic regulator AMP-activated protein kinase (AMPK) and/or REDD1 (regulated in DNA damage and development 1), which stop mTORC1 activity. Simultaneously, AMPK inhibits mTORC1 by phosphorylating Raptor [20].

#### 4.9.2. PTEN

In DLBCL, the phosphatase and tensin homolog (PTEN) is a critical negative regulator of PI3K/AKT signaling. By dephosphorylating phosphoinositide-3-phosphate, PTEN inhibits PI3K signaling (PIP3). PTEN deficiency causes PIP3 accumulation and, as a result, de-repression of the PI3K/AKT pathway, which promotes cell growth, proliferation, angiogenesis, and other cellular processes [95]. Several studies have suggested that PTEN loss/expression has different prognostic effects in DLBCL [96]. According to the Pfeifer et al. study, deregulation of the PI3K/AKT pathway by PTEN loss was detectable almost exclusively in 55 percent of GCB-DLBCL cases but only in 14 percent of non-GCB-DLBCL cases and poor prognosis in 248 primary DLBCL patients [97]. By contrast, another study conducted on two large cohorts of DLBCL showed that PTEN deletion and mutation can have only a slight effect on the clinical outcome of DLBCL patients. Thus, the study findings supported the hypothesis that the PI3K/PTEN/AKT and Myc signaling pathways are divergent rather than linear [96].

#### 4.9.3. MCT1

In order to maintain glycolysis, the constant expulsion of lactic acid from the cell, carried out by MCTs, is required. The MCT family has 14 members, although only four (MCT1, MCT2, MCT3, and MCT4) are found in the cell membrane and are classified as having the monocarboxylate and the H+ co-transportation systems. MCT1 and hypoxia-induced MCT4 are specialized in the co-transport of lactate and H+, especially for tumor and stromal cells. MCT1 is involved in both lactate extrusion and importation and was found to supply the tumor’s metabolic fuel via the transfer of metabolites between tumor cells and stromal cells [98]. In a previous study, a panel of glycolytic indicators was evaluated for their clinical and prognostic relevance in 104 B-cell lymphoma patients. In fact, the great majority of DLBCL samples for MCT1 (72.0%) and MCT4 (65.6%) were positive [2,15].

### 4.10. TMTV

There has been recent interest in the Total Metabolic Tumor Volume (TMTV) as a predictive marker in several lymphoma subtypes. TMTV uses FDG-PET/CT scans to measure the total tumor burden across the body. This measurement is based on segmenting each tumor uptake in whole-body acquisitions [99]. In DLBCL, baseline TMTV has been postulated as a predictive marker in many studies and has recently been verified by the PETAL trial analysis [100].

The combination of TMTV and tumor gene expression has shown intriguing prognostic utility by enabling patient risk classification. In a pharmacological study, TMTV was shown to impact rituximab exposure during first-line treatment, suggesting that it might be utilized to change the therapeutic index by employing a tailored dosage of rituximab. Interestingly, TMTV seems to be a potential prognostic biomarker, since a larger tumor volume is associated with a more severe cytokine release syndrome. However, further research is required to corroborate these conclusions [101].

## 5. The Novel and Future Therapeutic Aspect for B-Cell Lymphoma

The conventional treatment of B-cell lymphoma by chemoimmunotherapy has been used in the last decades, with varying outcomes according to either disease-related or patient-related factors. A significant percentage of different types of B-cell lymphomas experience relapse or refractoriness of the disease. This fact encouraged the expansion in trying to understand the intracellular process that is affected during lymphomagenesis, such as the signaling pathway, mitosis, and apoptosis, as well as the study of the microenvironment, which have helped in the development of new targeted therapies. Metabolic abnormalities may exist in a wide variety of lymphomas; studying and learning about them can lead to the identification of novel therapeutic targets, both alone and in combination with other therapies. Additionally, metabolomics approaches may be important for predicting outcomes and revealing new treatment options in the future [16,102]. In this context, we will explore novel treatment strategies for B-cell lymphomas from the metabolic perspective, as well as the correlation between metabolic dysfunction and immunotherapy response.

### 5.1. Energetic Pathways Targeting

mTORC1 encourages the flow across many metabolic pathways, including glycolysis, PPP, and OXPHOS. The targeting of mTORC1 and its metabolic downstream network may affect the generation of cellular energy and the viability of tumor cells. Considering the presence of tumor clusters OXPHOS (in a single-tumor entity) and the dependence of therapeutically resistant malignancies OXPHOS [103,104], combining rapalogs with oxidative metabolism inhibitors may improve patient responses. The biguanide metformin is the most well-known antimetabolic medication used for treating individuals with type II diabetes [105]. Metformin reduces mitochondrial complex I activity and, hence, decreases cell breathing, mitochondrial ATP generation, and glucose uptake [106,107]. Metformin-induced energy deprivation mechanistically blocks mTORC1 activity in the physiological [108] and pathological settings in an AMPK-dependent or independent way [109,110]. Metformin inhibits mTORC1 in human DLBCL and BL cell lines and decreases AMPK-dependent cell proliferation [111]. Therefore, the temsirolimus and metformin combination showed a better in vivo growth suppression than any therapy alone in B-lymphoma xenografts [111]. When glycolysis is inhibited, mTORC1 supports the viability of the cells by rescheduling the metabolism for glutaminolysis and metabolism of OXPHOS. MTORC1 inhibition reduces glycolysis independent of cell respiration. This avoids metabolic exhaust and synergistically impairs xenograft tumor growth [112]. Whereas LDH-A was a potential anticancer approach [32,113], specific lactate transport 1 (MCT1) inhibition of AZD3965 nonetheless leads to the glycolytic rate being negativized, thus providing an alternative strategy for MCT4-defective glycolytic tumors [114].

### 5.2. Amino Acids Targeting

Another potential treatment strategy that may be utilized in combination with rapalogs to further inhibit the mTORC1 function is to target extracellular sources of amino acids, which are indirectly detected by mTORC1 to control their activity. To date, arginase, leucine, and glutamine are the main amino acids implicated in mTORC1 regulation. The absorption of glutamine is needed to absorb leucine and stimulate lysosome-dependent leucine stimulation. L-asparaginase hydrolytes asparagine and glutamine extracellular (E-Coli, Erwinase, and other derivatives) and inhibits the activation of mTORC1 [17]. Glycolytic cancers upregulate GLS and move to glutamine metabolism following resistance to mTOR treatment [115]. The CB-839 GLS inhibitor inhibits glutamine conversion into glutamate and, therefore, restricts in vitro anaplerosis and cell proliferation of carbon-driven GLA sources needed by the TCA cycle [14,116,117,118]. More broadly, tumor cells that over-express MYC are very vulnerable to GLS, because they rely largely on glutamine oxidation to refill the TCA cycle, even under hypoxic circumstances [14,118]. This implies that the CB-839 mTOR inhibitor combination may be susceptible to patients with therapeutic MYC-translocated B-cell lymphomas. It must be noted that treatment methods that combine rapalogs and antimetabolic medicines are only beneficial if we can better describe the metabolic state of the tumor to address the best possible combination [20].

### 5.3. A New Therapeutic Approache from a Metabolic Perspective for B-Cell Lymphoma

#### 5.3.1. Metabolic Interventions with Immunotherapy

Immunotherapy is a recent therapeutic strategy for B-cell lymphoma, in which vaccinations are generated from patient-specific tumor antigens to enhance the immune response against tumors. Adoptive cellular therapy, immune checkpoint inhibitors, and novel antibody therapeutics are all now showing improvement in B-cell lymphoma [119]. In contrast, most patients do not benefit from immunotherapy due to inadequate reprogramming of the immunosuppressive tumor microenvironment (TME) and, hence, weak antitumor immunity. Various metabolic and nutrition-sensing pathways coordinate immune cell behaviors in response to TME nutrient availability. Notably, metabolic stress caused by tumor cell activity leads to decreased antitumor immune responses. Immunotherapy and targeted therapy are being used in combination with conventional B-cell lymphoma treatment regimens to enhance clinical outcomes. Due to inadequate cancer cell clearance, inherent characteristics are selected, resulting in metabolic vulnerabilities that are currently being studied for potential therapeutic strategies [120].

Targeting metabolic vulnerabilities in cancer cells is, therefore, an appealing therapeutic option. New evidence suggests that cancer cells may suppress antitumor immunity by competing for and depleting essential nutrients or by decreasing the metabolic fitness of tumor-infiltrating immune cells. Thus, metabolic treatments may improve immunotherapy efficacy. Importantly, the metabolic similarities between cancer and immune cells may limit synergistic effects. Targeting metabolic pathways that are necessary to both cancer cells and immune cells and that are altered by cancer cells to escape immunosurveillance holds great promise [121]. Furthermore, metabolic therapies may not only boost immune cell responses to highly immunogenetic tumors but also raise the immunogenicity of cancer cells, enhancing immunotherapy’s ability to treat a wider range of cancers (Table 2) [122].

#### 5.3.2. Metabolic Evaluation of the Immunomodulatory Therapy

Functional imaging, such as FDG-PET/CT, may reveal tumor lesions with high glucose metabolism, even if CT detects no major abnormalities in these lesions. In a few of instances, this resulted in a change in therapy for lymphoma patients who had been upstaged by the FDG-PET/CT assessment. As a biomarker for the early success or failure of R-CHOP in several trials, interim PET has been evaluated as a possible biomarker for those patients who will not benefit from first-line therapy. FDG-PET also plays an important role in the treatment of patients who are resistant to chemotherapy and may benefit from medicines with various mechanisms of action [101].

Immunotherapy, which is based on increasing the immune response to the tumor, is a very appealing treatment option for a variety of tumor types. Advances in immunomodulatory therapy, which have an influence on the interpretation of imaging, have necessitated the revision of the staging and response criteria for malignancy. The LYRIC criteria, proposed by Cheson et al., introduced a new response category, indeterminate response. This strategy incorporates the possibility of pseudo-progression reported with checkpoint inhibitors and also with immune modulators in general in order to avoid discontinuing a therapy that is actually effective and mandates additional biopsies or reimaging after 12 weeks [123]. Thus, novel FDG-PET/CT criteria are being created to evaluate the response to novel immunotherapy regimens and to serve as a more accurate predictive biomarker for predicting patient survival [101].

## 6. Conclusions

In conclusion, altered metabolism is already recognized as a biomarker of cancer, but still, our knowledge of cancer metabolism is evolving. Therefore, the discovery of metabolic biomarkers of B-cell lymphoma will improve the pretreatment stratification of patients and their progression and response to therapy. Biomarkers are intriguing not just for diagnostic purposes but also because they lead to the identification of novel therapeutic targets. Through this review, we found limited relevance on the use of metabolic biomarkers in the early diagnosis and prediction of B-cell lymphomas. Unfortunately, researchers have paid little attention to lymphoma metabolism. In contrast, there is a promising therapeutic significance for the use of metabolic markers to determine the appropriate treatment for lymphoma. Further molecular research in cell lines and animal models is necessary to establish that any or all of these markers may be used as indicators of the risk of B-cell lymphoma or may have other roles in earlier phases of carcinogenesis. Thus, treatment options for high-risk B-cell lymphoma subtypes might be improved by identifying distinct metabolic biomarkers for these subtypes.

## Figures and Tables

**Table 1:** Genetic mutations lead to B-cell lymphoma metabolisms.

Metabolism Influence	Genes	Ref.
Increase glycolysis and FAS	PI3K, mTOR	[42,72,73,74]
Reduces PPP activity and increases FAO	AMPK	[75,76,77,78]
Increase nucleotide biosynthesis	PRPS2	[79,80,81]
Organize glycolysis, TCA, glutamine, and proteins	MYC	[82,83]

**Table 2:** Clinical trials of metabolic interventions combined with immunotherapy.

Metabolic Therapy	Immunotherapy	Metabolic Target	Tumor Type	Study Phase	Status
Trigriluzole	Nivolumab or pembrolizumab	Glutamine and glutamate pathway inhibitors	Solid malignancies or lymphoma	II	Completed
CPI-006 (anti-CD73 antibody)	Pembrolizumab	Adenosine pathway inhibitors	Advanced-stage cancers, non-Hodgkin’s lymphoma	I	Recruiting

## Data Availability

Not applicable.
